# Luminescent Properties and Thermal Stability of [PPh_4_][Cu_3_I_4_] with a Unique Helical Structure

**DOI:** 10.3390/molecules30030543

**Published:** 2025-01-25

**Authors:** Luyi Chen, Andrey A. Petrov, Mingming Li, Sergey A. Fateev, Alexey B. Tarasov

**Affiliations:** 1 Department of Materials Science, Shenzhen MSU-BIT University, Shenzhen 518172, China; 2 Laboratory of New Materials for Solar Energetics, Department of Materials Science, Lomonosov Moscow State University, 1 Lenin Hills, 119991 Moscow, Russia

**Keywords:** hybrid halocuprates, hybrid iodocuprates, hybrid copper halides, tetraphenylphosphonium copper halide, luminescence, STE, high thermal stability

## Abstract

Hybrid halocuprates (I) with organic cations show great potential for optoelectronic applications due to their tunable luminescence and high thermal stability. In this study, the iodocuprate (I) [PPh_4_][Cu_3_I_4_], featuring unique helical chains of face-sharing tetrahedra, was synthesized and characterized. This compound exhibits a bandgap of 3.1 eV and orange luminescence at low temperature, attributed to self-trapped exciton emission. [PPh_4_][Cu_3_I_4_] demonstrates exceptional thermal stability among hybrid halocuprates with decomposition above 380 °C, forming a stable melt at ~255 °C without Cu^+^ oxidation in ambient atmosphere.

## 1. Introduction

Hybrid halocuprates (I) with organic cations represent a promising class of materials for advanced optoelectronic devices including photovoltaics, LEDs, and X-ray scintillating detectors [1]. These compounds offer tunable luminescent properties, high quantum yields, and non-toxicity, making them ideal for sustainable applications [1,2,3]. One of the advantages of hybrid halocuprates is their structural and compositional variety which governs a wide spectrum of optical properties, including bright luminescence, often coupled with intriguing properties like thermochromism [4,5,6,7,8,9] and mechanochromism [4,6].

The luminescent properties of halocuprates (I) with organic cations are known to be determined by their inorganic framework. In particular, room-temperature bright luminescence is usually observed for compounds with isolated units such as [CuX_2_]^−^, [CuX_3_]^2−^, [Cu_2_X_4_]^2−^, and [Cu_4_X_6_]^2−^, whereas for a chained framework, photoluminescence is usually weak or absent [10,11]. However, it was also found that some individual compounds with infinite [Cu_2_X_3_]^−^ and [Cu_3_X_4_]^−^ chains also demonstrate luminescence, and the actual influence of structural parameters on luminescence is yet to be revealed [12,13,14,15].

Although the crystal structures of alkylphosphonium halocuprates (I) were extensively explored in the 1980s by S. Jagner and H. Rartl with over a dozen compounds discovered [16,17,18,19,20,21,22,23], it was found only recently that similarly to alkylammonium halocuprates, such compounds demonstrate bright luminescence originating from self-trapped exciton emission (STE) with high quantum yields approaching 100% [24,25,26,27,28,29,30,31,32].

Several luminescent compounds with tetraphenylphosphonium (PPh_4_^+^) cations have been reported so far. While the structures [PPh_4_]_2_[Cu_2_I_4_], [PPh_4_]_2_[Cu_4_I_6_]·2(CH_3_)_2_CO, and [PPh_4_]_2_[Cu_4_I_6_]·2DMSO with isolated planar dimer [Cu_2_X_4_]^2−^ and [Cu_4_X_6_]^2−^ cluster units expectedly demonstrated bright luminescence with PLQY = 99.5% for the latter compound [6,24,33], structures with isolated [CuX_2_]^−^ units (PPh_4_CuX_2_, X = Br, Cl) showed weak luminescence [34], whereas structures with infinite [Cu_3_I_4_]^−^ chains [PPh_3_R][Cu_3_I_4_] (R = Me, Et, Bu) also turned out to be luminescent [12]. Furthermore, many hybrid halocuprates (I) with alkylphosphonium cations demonstrated strong coupling between polymorphism and optical properties (e.g., for [PPh_4_]_2_[Cu_2_I_4_] with four polymorph modifications [6,35] and α/β-[PPh_3_Me]_2_[Cu_4_I_6_] [36]), which complicates the prediction of the luminescent behavior of materials based on their structure even further.

Apart from structural influence acting as a structure-directing agent, phosphonium cations that include aromatic substituents can also participate in the formation of the upper valence band (HOMO) and the conduction band (LUMO). In particular, phenyl rings and the phosphorus atom itself can introduce new electronic states, shifting the energy levels of the system, which can directly impact the bandgap and luminescent properties of materials [25,37]. Moreover, such cations may also take part in charge transfer processes upon excitation, changing the nature and probability of radiative transitions [36,38].

Whereas two polymorphs [PPh_4_]_2_[Cu_2_I_4_] with isolated dimers [Cu_2_I_4_]^2−^ were recently found to be luminescent [6], the properties of [PPh_4_][Cu_3_I_4_] remain unknown. Among all known hybrid halocuprates, this compound exhibits a unique structure composed of helical chains of face-sharing tetrahedra [39], while this distinct structural arrangement highlights the need for an in-depth investigation of [PPh_4_][Cu_3_I_4_]’s optical properties to understand its potential as a functional material.

In this article, we present for the first time the optical absorption, luminescent properties, and thermal stability of [PPh_4_][Cu_3_I_4_] and compare it with similar iodocuprate (I) phases.

## 2. Results and Discussion

**Figure 1:**
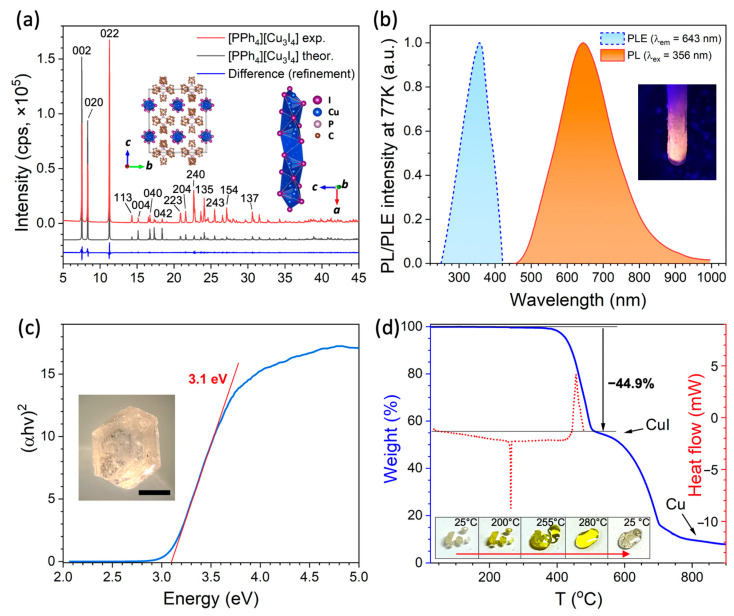
(**a**) X-ray diffraction pattern of [PPh_4_][Cu_3_I_4_], (**b**) PLE and PL spectra measured at 77 K, (**c**) absorption spectrum in Tauc coordinates and inset with photo of crystal (scalebar is 0.3 mm), (**d**) TG/DSC curves with inset image of melting crystals.

Transparent crystals of [PPh_4_][Cu_3_I_4_] were synthesized by cooling a saturated solution of [PPh_4_]I and CuI (1:3 molar ratio) in acetonitrile. The diffraction pattern (Figure 1a) of the powdered crystals matches well with the structure reported by Hartl et al. in 1992 [39]. The profile analysis confirmed that [PPh_4_][Cu_3_I_4_] crystallizes in an orthorhombic *Ccce* space group with parameters a = 11.5821(5) Å, b = 21.1227(9) Å, and c = 23.3228(9) Å.

Structurally, [PPh_4_][Cu_3_I_4_] is similar to other hybrid halocuprates like ACu_3_X_4_ and ACu_2_X_3_ (A = organic cation; X = I, Br, Cl) which consist of infinite chains of edge- and face-sharing CuI_4_ tetrahedra. However, no other halocuprates with only the face-sharing connectivity of CuX_4_ tetrahedra have been reported. Many ACu_2_I_3_ iodocuprates with a 1D [Cu_2_I_3_]^−^ chain have been reported [13], but fewer ACu_3_I_4_ structures were found, namely three structures [PPh_3_R][Cu_3_I_4_] (R = Me, Et, Bu) [12], [bp4][Cu_3_I_4_] (bp4 = N-methyl-4,4′-bipyridinium) [40], and [EtS-4-C_5_H_4_NEt][Cu_3_I_4_] [41], all with infinite [Cu_3_I_4_]^−^ chains of edge-sharing tetrahedra, [(DodecylMe_2_S)[(DodecylMeS)_3_)][Cu_3_I_4_] with isolated [Cu_3_I_4_]^−^ (also with edge-sharing structure) stabilized by sulfur atoms [42], and [N(CH_3_CH_2_CH_2_)_4_][Cu_3_I_4_] with 1D chains made of edge-sharing units of three face-sharing tetrahedra [23,43]. Notably, no bromide structures [Cu_3_Br_4_]^−^ have been found so far, which agrees with Pauling’s third rule, predicting the lower connectivity of CuBr_4_ polyhedra due to their smaller radius and polarizability [44].

The Cu-Cu distance in [PPh_4_][Cu_3_I_4_] measured from the crystal structure data is as small as 2.30 Å due to the face-shared structure of the helical [Cu_3_I_4_]^−^ chains, suggesting strong Cu–Cu bonding interactions [39]. In contrast, the chains themselves are loosely packed compared to the majority of structures demonstrating hexagonal packaging. PPh_4_^+^ cations are also not tightly packed in the structure and do not form the π-π stacking of phenyl groups as was observed for [PPh_3_Et][Cu_3_I_4_]. It can be concluded therefore that the cations are likely to only produce templating effects with a weak influence on optoelectronic properties.

We found that at room temperature, [PPh_4_][Cu_3_I_4_] does not exhibit luminescence unlike [PPh_3_R][Cu_3_I_4_] (R = Me, Et, Bu) and other compounds with PPh_4_^+^ cations ([PPh_4_]_2_[Cu_2_I_4_], [PPh_4_]_2_[Cu_4_I_6_]·2OC(CH_3_)_2_, [PPh_4_]_2_[Cu_4_I_6_]·2DMSO) [12,24,33]. However, at 77 K (−196 °C), it demonstrates orange emission attributed to STE with a PL maximum at 643 nm (1.93 eV) corresponding to CIE color coordinates of (0.33, 0.36). An excitation peak was observed at 356 nm (3.48 eV), with a Stokes shift of 287 nm (1.55 eV) and an FWHM of 177 nm. (Figure 1b). The absence of luminescence at room temperature may be attributed to the small Cu-Cu distance (2.3 Å) in the face-sharing tetrahedra structure and effective charge transfer between the excited and unexcited states resulting in a quenching effect. Such a shortening of the Cu-Cu distance was suggested to be the reason for luminescence quenching in Cu_6_I_6_·2HMTA (HMTA = hexamethylenetetramine) crystals upon the partial substitution of Cu to Ag [45]. However, although the Cu-Cu distance was shown to be one of the main factors governing luminescence and responsible for thermochromism [46], the example of [TMA][Cu_2_I_3_] (TMA = tetramethylammonium) with a small 2.34 Å Cu-Cu distance and luminescence at room temperature indicates that other structural features such as interactions with organic cations also contribute to luminescent properties [13].

The optical bandgap of [PPh_4_][Cu_3_I_4_] is determined to be E_g_ = 3.10 eV, based on the diffuse reflectance spectrum plotted in Tauc coordinates (Figure 1c), and it matches well with the photoluminescence excitation spectrum with an onset at ~3.02 eV. This bandgap is similar to the values of [PPh_3_R][Cu_3_I_4_] (~2.9 eV for R = Me, Et, n-Bu) [12], α/β-[TMA][Cu_2_I_3_] (3.1/3.3 eV, TMA = tetramethylammonium) [13], and [NMP][Cu_2_I_3_] (2.85 eV, NMP = N-methylpyridinium) [14] and slightly lower than that for ACu_2_I_3_ iodocuprates such as [MA][Cu_2_I_3_] (3.62 eV, MA = methylammonium) [47], [TMS][Cu_2_I_3_] (3.54 eV, TMS = trimethylsulfonium), and [TMSO][Cu_2_I_3_] (3.63 eV, TMS = trimethylsulfoxonium) [48]. This observation agrees with the general trend that a lower degree of [CuX_4_] tetrahedra condensation results in a higher bandgap [10,11,49].

The low-temperature luminescence spectrum of [PPh_4_][Cu_3_I_4_] with PL_max_ = 643 nm is similar to that for other iodocuprates with infinite [Cu_3_I_4_]^−^ and [Cu_2_I_3_]^−^ chains. In particular, also non-emissive at room temperature, at 77 K, [MA]Cu_2_I_3_ and [TMS]Cu_2_I_3_ have yellow-orange luminescence with PL_max_ = 602 nm and PL_max_ = 660 nm, respectively [47,50]; [NMP][Cu_2_I_3_] has PL_max_ = 700 nm [14]; and [PPh_3_R][Cu_3_I_4_] has an active emission band at low temperature centered at ~580–600 nm [12].

TG/DSC measurements showed the excellent thermal stability of [PPh_4_][Cu_3_I_4_] with decomposition above 380 °C (Figure 1d) which is one of the highest among hybrid halocuprates [12,27,38,40]. The first step of weight loss is attributed to the loss of PPh_4_I and the formation of CuI which perfectly matches with the calculated value (44.9%), whereas in the second stage, it decomposes with some remains of Cu. DSC measurement shows an endo-effect at ~255 °C which corresponds to melting. Notably, upon melting, a stable, transparent light yellow liquid is formed (Figure 1d, inset) without the oxidation of Cu^+^ to Cu^2+^ unlike many other halocuprates (I) [10]. Upon cooling below 190 °C, it crystallizes spontaneously without signs of the glass transition, as was observed for some 0D iodocuprates such as [PPh_3_Me]_2_[Cu_4_I_6_] [36].

## 3. Materials and Methods

### 3.1. Synthesis of Crystals

A total of 387.7 mg of tetraphenylphosphonium iodide (98%, anhydrous, Macklin, Shanghai, China) and 777.2 mg of copper (I) iodide (99%, Macklin) were dissolved in a 1:3 molar ratio in 5 mL of acetonitrile (99.8%, anhydrous, Sigma Aldrich, Saint Louis, MO, USA) in a glovebox with an Ar atmosphere. The solution was stirred at 70 °C until the precursors completely dissolved. After the addition of 20 µL of H_3_PO_2_ to prevent Cu^+^ oxidation, the solution became transparent. Then, a hot solution was filtered through a PTFE syringe filter (0.45 µm) and left to cool (~0.5°/min) to room temperature. After 24 h, transparent crystals of [PPh_4_][Cu_3_I_4_] were collected and dried on a filter paper.

### 3.2. Measurements

PL and PLE spectra were recorded at 77 K using a HORIBA QuantaMaster 8000 spectrofluorometer (Kyoto, Japan) from [PPh_4_][Cu_3_I_4_] crystals ground in an Ar atmosphere sealed in an NMR quartz tube. The maximum in the PLE spectrum (356 nm) was chosen as the excitation wavelength for measuring the PL spectrum. Excitation spectra were recorded at a wavelength corresponding to the position of the maximum of the PL spectrum (643 nm). A background was subtracted from the obtained spectra, and the resulting data were smoothed.

A diffuse reflectance spectrum (DRS) was recorded from [PPh_4_][Cu_3_I_4_] powder crystals at room temperature on a PerkinElmer Lambda 950 spectrophotometer (Springfield, IL, USA) at 25 °C in the wavelength range from 250 to 650 nm.

Powder X-ray diffraction patterns were obtained on a Rigaku Smartlab SE diffractometer (Kyoto, Japan) using radiation from a Cu anode and recording the CuKa component via a 2D detector in the Bragg–Brentano geometry in the 2θ range of 5–60° with a step of 0.02°. Crystal lattice parameters were refined from powder diffraction patterns using Jana 2006 software (version 20/02/2023) [51]. The visualization and analysis of the crystal structure were carried out using VESTA software (ver. 3.4.7) [52].

Thermogravimetric measurements were conducted using a Mettler Toledo TGA 2 system (Greifensee, Switzerland) in the temperature range of 25–900 °C. A small crystal of [PPh_4_][Cu_3_I_4_] was placed in an alumina crucible with a pierced lid and measured in a flow of Ar (30 mL/min) at a scanning speed of 5 °C/min.

A differential scanning calorimetry curve was obtained on a Mettler Toledo DSC 3 device (Mettler-Toledo, Greifensee, Switzerland) in the temperature range 25–500 °C. A small crystal of [PPh_4_][Cu_3_I_4_] was placed in an aluminum crucible with a pierced lid and measured in a flow of Ar (30 mL/min) at a scanning speed of 5 °C/min.

## 4. Conclusions

In this work, we synthesized and investigated the optical properties and thermal stability of [PPh_4_][Cu_3_I_4_], a hybrid iodocuprate (I) with a unique helical chain structure. This compound was synthesized as transparent crystals and confirmed to have an orthorhombic crystal structure. Optical studies revealed a bandgap of 3.10 eV and low-temperature luminescence with an emission peak at 643 nm, attributed to self-trapped excitons. Thermal analysis showed one of the highest stabilities among hybrid halocuprates, with decomposition temperatures exceeding 380 °C and a melting point at ~255 °C, forming a stable liquid. This study provides valuable insights into the relationship between structure and luminescent properties, contributing to a deeper understanding of hybrid halocuprates.

## Data Availability

Data are contained within the article.

## References

[1] Banerjee D., Saparov B. (2023). Ultrabright Light Emission Properties of All-Inorganic and Hybrid Organic-Inorganic Copper(I) Halides. Chem. Mater..

[2] Zhang P., Yan Z., Li C., Du Y., Ma L., Wang Z., Lin T., Zhao L., Xiao J. (2024). Low-dimensional hybrid copper(I) halides single crystals: Synthesis, structures, and tunable photoluminescence. Chem. Eng. J..

[3] Hamdi I., Khan Y., Aouaini F., Seo J.H., Koo H.-J., Turnbull M.M., Walker B., Naïli H. (2022). A copper-based 2D hybrid perovskite solar absorber as a potential eco-friendly alternative to lead halide perovskites. J. Mater. Chem. C.

[4] Liu X., Zhang T., Zhou L., Li M., He R. (2024). Dual-Emissive γ-[Cu_4_I_8_]^4−^ Enables Luminescent Thermochromism in an Organic–Inorganic Hybrid Copper(I) Halide. Inorg. Chem..

[5] Yang Z.-C., Song K.-Y., Zhou P.-K., Zong L.-L., Li H.-H., Chen Z.-R., Jiang R. (2022). Sensitive luminescence mechanochromism and unique luminescence thermochromism tuned by bending the P–O–P skeleton in the diphosphonium/iodocuprate(I) hybrid. CrystEngComm.

[6] Thefioux Y., Cordier M., Massuyeau F., Latouche C., Martineau-Corcos C., Perruchas S. (2020). Polymorphic Copper Iodide Anions: Luminescence Thermochromism and Mechanochromism of (PPh_4_)_2_[Cu_2_I_4_]. Inorg. Chem..

[7] Maderlehner S., Leitl M.J., Yersin H., Pfitzner A. (2015). Halocuprate(I) zigzag chain structures with N-methylated DABCO cations—Bright metal-centered luminescence and thermally activated color shifts. Dalt. Trans..

[8] Li S.-L., Zhang F.-Q., Zhang X.-M. (2015). An organic-ligand-free thermochromic luminescent cuprous iodide trinuclear cluster: Evidence for cluster centered emission and configuration distortion with temperature. Chem. Commun..

[9] Dev A.V., Basavarajappa M.G., Deshpande S.S., Mukherjee P., Ajayakumar A., Muthu C., Okamoto T., Chakraborty S., Sarma D.D., Biju V. (2024). Thermally Induced Reversible Fluorochromism by Self-Trapped Excitonic Emission in a Two-Dimensional Hybrid Copper(I)-Halide Single Crystal. Chem. Mater..

[10] Belikova D.E., Fateev S.A., Khrustalev V.N., Kozhevnikova V., Ordinartsev A.A., Dzuban A.V., Goodilin E.A., Tarasov A.B. (2024). Bright luminescence of new low-melting copper(I) chlorides with compact organic cations. J. Mater. Chem. C.

[11] Belikova D.E., Fateev S.A., Khrustalev V.N., Marchenko E.I., Goodilin E.A., Wang S., Tarasov A.B. (2023). Exceptional structural diversity of hybrid halocuprates(I) with methylammonium and formamidinium cations. Dalt. Trans..

[12] Zhang W.T., Liu J.Z., Liu J.B., Song K.Y., Li Y., Chen Z.R., Li H.H., Jiang R. (2018). Quaternary Phosphorus-Induced Iodocuprate(I)-Based Hybrids: Water Stabilities, Tunable Luminescence and Photocurrent Responses. Eur. J. Inorg. Chem..

[13] Li S.L., Zhang R., Hou J.J., Zhang X.M. (2013). Photoluminescent cuprous iodide polymorphs generated via in situ organic reactions. Inorg. Chem. Commun..

[14] Yue C.-Y., Lin N., Gao L., Jin Y.-X., Liu Z.-Y., Cao Y.-Y., Han S.-S., Lian X.-K., Hu B., Lei X.-W. (2019). Organic cation directed one-dimensional cuprous halide compounds: Syntheses, crystal structures and photoluminescence properties. Dalt. Trans..

[15] Artem’ev A.V., Berezin A.S., Taidakov I.V., Bagryanskaya I.Y. (2020). Synthesis of dual emitting iodocuprates: Can solvents switch the reaction outcome?. Inorg. Chem. Front..

[16] Hartl H., Brüdgam I., Mahdjour-Hassan-Abadi F. (1985). Synthese und Strukturuntersuchungen von Iodocupraten(I) VI. Iodocuprate(I) mit zweikernigen Anionen [Cu_2_I_4_]^2−^ und [Cu_2_I_6_]^4−^. Z. Naturforsch. B.

[17] Andersson S., Jagner S., Grenthe I., Salvatore F., Niinistö L., Volden H.V., Weidlein J., Zingaro R.A. (1986). Anionic Configurations and Ligand Concentrations in Butyltriphenylphosphonium Dibromocuprate(I) and Bis(butyltriphenylphosphonium) hexa-mu-bromo-tetrahedro-tetracuprate(I). Acta Chem. Scand..

[18] Andersson S., Jagner S., Kjekshus A., Salvatore F., Niinistö L. (1987). Coordination of Copper(I) in bis(tetramethylphosphonium) Tribromocuprate(I) and bis(tetraethylphosphonium) Di-mu-bromo-dibromodicuprate(I); Comparison of Anionic Configurations in Bromocuprates(I) Crystallizing with Symmetrical Tetraalkylammonium and Rela. Acta Chem. Scand..

[19] Andersson S., Jagner S., Popik M.V., Sadova N.I., Shaieb D., Haaland A., Lappert M.F., Leung W.-P., Rypdal K. (1988). Coordination of Copper(I) in Two Novel Chlorocuprate(I) Anions; Structures of Tetramethylphosphonium Catena-mu-Chloro-mu3-chloro-[mu-chloro-dicuprate(I)] and Bis(tetramethylphosphonium) Trichlorocuprate(I). Acta Chem. Scand..

[20] Andersson S., Jagner S., Sykes A.G., Leskelä M., Hoyer E. (1985). Crystal Structures of Tetraphenylarsonium Dichlorocuprate(I), Tetraphenylphosphonium Dichlorocuprate(I) and Tetraphenylphosphonium Dibromocuprate(I). Acta Chem. Scand..

[21] Andersson S., Jagner S., Østvold T., Tammenmaa M., Volden H.V. (1985). Crystal Structure of Ethyltriphenylphosphonium Dibromocuprate(I), [P(C_2_H_5_)(C_6_H_5_)_3_][CuBr_2_]. Acta Chem. Scand..

[22] Andersson S., Jagner S., Lehmann M.S., Tammenmaa M., Volden H.V. (1985). Crystal Structure of Propyltriphenylphosphonium Dibromocuprate(I), [P(C_3_H_7_)(C_6_H_5_)_3_][CuBr_2_]. Acta Chem. Scand..

[23] Hartl H., Mahdjour-Hassan-Abadi F. (1984). Synthese und Strukturuntersuchungen von Iodocupraten(I), III. Iodocuprate(I) mit isolierten Ketten ^1^_∞_[Cu_2_I_3_]^−^ bzw. ^1^_∞_[Cu_3_I_4_]^−^. Z. Naturforsch. B.

[24] Chen K., Chen B., Xie L., Li X., Chen X., Lv N., Zheng K., Liu Z., Pi H., Lin Z. (2024). Organic–Inorganic Copper Halide Compound with a Near-Unity Emission: Large-Scale Synthesis and Diverse Light-Emitting Applications. Adv. Funct. Mater..

[25] Wu J., Qi J.L., Guo Y., Yan S., Liu W., Guo S.P. (2023). Reversible tri-state structural transitions of hybrid copper(i) bromides toward tunable multiple emissions. Inorg. Chem. Front..

[26] Li D.-Y.Y., Wu J.-H.H., Wang X.-Y.Y., Zhang X.-Y.Y., Yue C.-Y.Y., Lei X.-W.W. (2023). Reversible Triple-Mode Photo- and Radioluminescence and Nonlinear Optical Switching in Highly Efficient 0D Hybrid Cuprous Halides. Chem. Mater..

[27] Cao S., Lai J., Wang Y., An K., Jiang T., Wu M., He P., Feng P., Tang X. (2025). Vacuum-filtration fabrication of copper-based halide scintillation screen for high-resolution X-ray imaging. J. Lumin..

[28] Xie L., Liu Z., Yang H., Chen K., Lv N., Pi H., Chen X., Li X., Liu Z., Li S. (2024). Ultra Broad-Band Excitable Organic–Inorganic Copper(I) Halides: Large-Scale Synthesis, Outstanding Stability, and Highly Efficient White Light-Emitting Diodes Application. Adv. Opt. Mater..

[29] Fu P., Geng S., Mi R., Wu R., Zheng G., Su B., Xia Z., Niu G., Tang J., Xiao Z. (2024). Achieving Narrowed Bandgaps and Blue-Light Excitability in Zero-Dimensional Hybrid Metal Halide Phosphors via Introducing Cation–Cation Bonding. Energy Environ. Mater..

[30] Popy D.A., Singh Y., Tratsiak Y., Cardoza A.M., Lane J.M., Stand L., Zhuravleva M., Rai N., Saparov B. (2024). Stimuli-responsive photoluminescent copper(I) halides for scintillation, anticounterfeiting, and light-emitting diode applications. Aggregate.

[31] Lai J., Li C., Wang Z., Guo L., Wang Y., An K., Cao S., Wu D., Liu Z., Hu Z. (2024). Photoluminescence-Tunable organic phosphine cuprous halides clusters for X-ray scintillators and white light emitting diodes. Chem. Eng. J..

[32] Zhang X., Yang H.J., Mao Y.J., Wang J.Y., Pang A., Xu L.J. (2024). Structural regulation and photophysical insights into zero-dimensional organic copper(I) halides with thiophene-substituted phosphonium cation. J. Mol. Struct..

[33] Jalilian E., Liao R.Z., Himo F., Brismar H., Laurell F., Lidin S. (2011). Luminescence properties of the Cu_4_I_6_^2−^ cluster. CrystEngComm.

[34] Popy D.A., Creason T.D., Zhang Z., Singh D.J., Saparov B. (2022). Electronic structures and optical properties of (Ph_4_P)MX_2_ (M = Cu, Ag; X = Cl, Br). J. Solid State Chem..

[35] Pfitzner A., Schmitz D. (1997). Two New Modifications of [P(C_6_H_5_)_4_]_2_[Cu_2_I_4_]. Z. Anorg. Allg. Chem..

[36] Li B., Jin J., Liu X., Yin M., Zhang X., Xia Z., Xu Y. (2024). Multiphase Transformation in Hybrid Copper(I)-Based Halides Enable Improved X-ray Scintillation and Real-Time Imaging. ACS Mater. Lett..

[37] Lin N., Wang R.C., Zhang S.Y., Lin Z.H., Chen X.Y., Li Z.N., Lei X.W., Wang Y.Y., Yue C.Y. (2023). 0D Hybrid Cuprous Halide as an Efficient Light Emitter and X-Ray Scintillator. Laser Photonics Rev..

[38] An R., Wang Q., Liang Y., Du P., Lei P., Sun H., Wang X., Feng J., Song S., Zhang H. (2025). Reversible Structural Phase Transitions in Zero-Dimensional Cu(I)-Based Metal Halides for Dynamically Tunable Emissions. Angew. Chem. Int. Ed..

[39] Hartl H., Mahdjour-Hassan-Abadi F. (1995). Synthesis and Structure Investigations of Iodocuprates(I). Part 16. ((C_6_H_5_)_4_P)^1^_∞_(Cu_3_I_4_)—The First Compound with a Helical Chain of Face-Sharing Tetrahedra as a Structural Element. ChemInform.

[40] Adam D., Herrschaft B., Hartl H. (1991). Synthese und Strukturuntersuchungen von Iodocupraten(I)/Synthesis and Structure Investigations of Iodocuprates(I). Z. Naturforsch. B.

[41] Cheng J.-K.K., Yao Y.-G.G., Zhang J., Li Z.-J.J., Cai Z.-W.W., Zhang X.-Y.Y., Chen Z.-N.N., Chen Y.-B.B., Kang Y., Qin Y.-Y.Y. (2004). A Simultaneous Redox, Alkylation, Self-Assembly Reaction under Solvothermal Conditions Afforded a Luminescent Copper(I) Chain Polymer Constructed of Cu_3_I_4_^−^ and EtS-4-C_5_H_4_N^+^Et Components (Et = CH_3_CH_2_). J. Am. Chem. Soc..

[42] Paulsson H., Berggrund M., Fischer A., Kloo L. (2004). Novel Layered Structures Formed by Iodocuprate Clusters Stabilized by Dialkylsulphide Ligands. Z. Anorg. Allg. Chem..

[43] Jalilian E., Lidin S. (2011). Size matters—Sometimes. The [Cu_x_I_y_]_(y−x)_^−^(NR_4_)^+^_(y−x)_ systems. CrystEngComm.

[44] Burdett J.K., McLarnan T.J. (1982). An orbital explanation for Pauling’s third rule. J. Am. Chem. Soc..

[45] Chen L., Dong X., Mo Z., Wang H., Ye J., Zhang K., Chen X. (2021). Efficient Restraint of Intra-Cluster Aggregation-Caused Quenching Effect Lighting Room-Temperature Photoluminescence. Adv. Opt. Mater..

[46] Kim T.H., Shin Y.W., Jung J.H., Kim J.S., Kim J. (2008). Crystal-to-Crystal Transformation between Three Cu I Coordination Polymers and Structural Evidence for Luminescence. Angew. Chem. Int. Ed..

[47] Petrov A.V.A., Fateev S.A., Grishko A.Y., Ordinartsev A.A., Petrov A.V.A., Seregin A.Y., Dorovatovskii P.V., Goodilin E.A., Tarasov A.B. (2021). Optical properties and electronic structure of methylammonium iodocuprate as an X-ray scintillator. Mendeleev Commun..

[48] Pinky T., Popy D.A., Zhang Z., Jiang J., Pachter R., Saparov B. (2024). Synthesis and Characterization of New Hybrid Organic–Inorganic Metal Halides [(CH_3_)_3_SO]M_2_I_3_ (M = Cu and Ag). Inorg. Chem..

[49] Marchenko E.I., Fateev S.A., Goodilin E.A., Tarasov A.B. (2022). Band Gap and Topology of 1D Perovskite-Derived Hybrid Lead Halide Structures. Crystals.

[50] Petrov A.A., Khrustalev V.N., Zubavichus Y.V., Dorovatovskii P.V., Goodilin E.A., Tarasov A.B. (2018). Synthesis and crystal structure of a new hybrid methylammonium iodocuprate. Mendeleev Commun..

[51] Petříček V., Dušek M., Palatinus L. (2014). Crystallographic Computing System JANA2006: General features. Z. Krist. Mater..

[52] Momma K., Izumi F. (2011). VESTA 3 for three-dimensional visualization of crystal, volumetric and morphology data. J. Appl. Crystallogr..

